# Climate change, migration and health systems resilience: Need for interdisciplinary research

**DOI:** 10.12688/f1000research.17559.2

**Published:** 2019-04-01

**Authors:** Valéry Ridde, Tarik Benmarhnia, Emmanuel Bonnet, Carol Bottger, Patrick Cloos, Christian Dagenais, Manuela De Allegri, Ariadna Nebot, Ludovic Queuille, Malabika Sarker

**Affiliations:** 1 IRD (French Institute For Research on Sustainable Development), CEPED (IRD-Université Paris Descartes), Universités Paris Sorbonne Cités, ERL INSERM SAGESUD, Paris, France; 2 University of Montreal Public Health Research Institute (IRSPUM), 7101 Avenue du Parc, Room 3060, Montreal, QC, Canada; 3 l’Institut Français des Migrations, Paris, France; 4 Department of Family Medicine and Public Health & Scripps, Institution of Oceanography, University of California, California, USA; 5 IRD (French Institute For Research on Sustainable Development), UMI Résiliences 236, Bondy, France; 6 School of Public Health, University of Montreal (ESPUM), 7101 Avenue du Parc, Montreal, QC, Canada; 7 School of Social Work, Faculty of Arts and Sciences, University of Montreal, Montreal, QC, Canada; 8 Department of Psychology, University of Montreal, Montreal, Canada; 9 Heidelberg Institute of Global Health, Medical Faculty, Heidelberg University, Heidelberg, Germany; 10 Independent Consultant, Pharmacist and Public Health Specialist, Paris, France; 11 Pan American Health Organization, Haiti Office, Port-au-Prince, Haiti; 12 BRAC James P Grant School of Public Health, BRAC University, Dhaka, Bangladesh

**Keywords:** Climate Change, Migrations, Health Systems, Resilience, Interdisciplinary

## Abstract

Climate change is one of today's major challenges, and among the causes of population movement and international migration. Climate migrants impact health systems and how their ability to respond and adapt to their needs and patterns.  To date, the resilience of health systems in the context of climate change has barely been explored.

The purpose of this article is to show the importance of studying the relationship between climate change, migration, and the resilience of health systems from an interdisciplinary perspective.

Resilience is an old concept, notably in the field of psychology, and is increasingly applied to the study of health systems. Yet, no research has analysed the resilience of health systems in the context of climate change. While universal health coverage is a major international goal, little research to date focused on the existing links between climate, migration, health systems and resilience.

We propose an interdisciplinary approach relying on the concept of health system resilience to study adaptive and transformative strategies to articulate climate change, migration and health systems.

## Introduction

*“Four thousand migrants arrive in Dhaka, the capital of Bangladesh due to various ‘push’ factors including frequent natural disasters*” ^1^. Environmental changes due to climate change are projected to cause substantial increases in population movement, within and between countries, in the coming decades. Haiti faces a similar situation according to a 2008 report it is estimated that 100,000 people have moved for climate change reasons from rural areas to the capital Port-au-Prince ^2^. Environmental changes (e.g. drought, soil erosion, extreme weather events, etc.) lead to substantial impacts on health, economic and political dimensions at the population level, including influencing migration patterns and may result in adverse health outcomes, both for displaced and for host populations ^3– 5^. The World Health Organization (WHO) consistently identifies climate change as a defining challenge of the 21 ^st^ century; and considers it an emerging priority for the public health community to ensure protection against its health impact ^6, 7^. In 2015, The Rockefeller Foundation and *The Lancet* published the report of the Commission on Planetary Health ^8^ and the UN Sustainable Development Goal 13 calls for “urgent action to combat climate change and its impacts".

For this article, we conducted a heuristic non-systematic literature review on climate change, migration and health systems. As a result of a peer-reviewed article search in the PUBMED database using climate change, health systems, and migrants as keywords, only 10 results published between 1994 and 2017 were identified. Of these, six (60%) were written in the past decade and included: two opinion papers, two study reviews, one qualitative study, and one protocol for a review that will be completed in 2018.

In this article, we describe and discuss the fundamental role that health care systems resilience can play in this regard and we identify interdisciplinary research as key to better understanding the existing linkages between climate change, migration and health systems and how to build more resilient health systems. We also propose some questions and axes to orient future research proposals.

## Climate migrants and health challenges

Climate change can be translated to many forms of environmental degradations, including hurricanes ^9^, rising sea levels, and/or reduced rainfall in drylands and water scarcity ^10^. Populations confronted by climate change consequences such as exposure to hazards, loss in land productivity, absence of habitability, and/or shortage of food/energy/water security may have difficulties to subsist in a given area ^11^. Climate change consequences compounded by socio-economic pressures and/or political instability, increase propensity to migrate. Although evidence is still missing to prove this association, environmental factors are increasingly influencing a complex pattern of human mobility. A recent paper suggests “ *a statistically significant relationship between fluctuations in asylum applications and weather anomalies*” ^12^. Climate migrants may be forced to leave their homes due to rapid-onset disasters, such as flooding and hurricanes (as in Haiti and Bangladesh for example) ^1, 2, 13, 14^.

Nowadays, there is no conceptual consensus on the notions of environmental refugee or climate change migrants yet, or the more rarely used terms ecomigrants or environmentally displaced persons ^15, 16^. Since 2007, the International Organization for Migration (IOM) has defined environmental migrants as “ *persons or groups of persons who, for compelling reasons of sudden or progressive change in the environment that adversely affects their lives or living conditions, are obliged to leave their habitual homes, or choose to do so, either temporarily or permanently, and who move either within their country or abroad*” ^17^. Others suggest restricting the definition to victims of extreme weather, drought/water scarcity, and sea-level rise and excluding the effects of the spread of tropical diseases ^16^. The simple fact is that the implications climate change are unknown will bear on the distribution of the world population ^18^. Current estimates range between 25 million and 1 billion people by 2050. and according to the 2017 Lancet Countdown report “ *the total number of people vulnerable to migration might increase to 1 billion by the end of the century without significant further action on climate change*” ^5^.

Climate-related migrants may or may not perceive how climate change influences and has an impact on their health needs and social patterns. For example, in Burkina Faso, the close relationship between climate change and flooding is not always fully perceived by the Burkina population suffering from it, as documented by the authors of this manuscript in previous studies. ( Box 1) However, climate-related migrants experience difficulties or face challenges similar to those of refugees fleeing war and/or political persecution: overcrowded settlements, unsanitary conditions, poor nutritional status, unsafety, inequity and limited access to health services ^1, 2, 19, 20^. Although these migrants may experience similar situations with regard to their health and access to healthcare research has focused almost exclusively on the latter rather than on the former. In addition, environmental change migrant population are usually the most vulnerable because migration is often expensive and climate change factors can easily be in addition to other strong socio-economic factors. For example, Haiti and Bangladesh were respectively ranked 3 ^rd^ and 6 ^th^ globally in the Long-Term Climate Risk Index (CRI) from 1995 to 2014 ^21^, while their health systems’ performance were ranked by the WHO in 2000 as 138 ^th^ and 88 ^th^, respectively, out of 191 countries ^22^. The very recent Global Climate Risk Index 2018 confirms Haiti and Bangladesh as high risk countries but also shows that several African countries (Mozambique, Malawi, Ghana, Madagascar) are highly affected and have little research on climate migrants ^23^.

Box 1. **Local perception about the link between climate change and flooding by displaced population in Burkina Faso**

A recent survey of Sahelian floods in Ouagadougou, Burkina Faso ^24^, reveals that climate change is not perceived by the population as being responsible for the floods. They consider that the responsibility lies more with the authorities who did not act to maintain the water supply facilities. The links with climate change do not seem to be perceived by the citizens of Ouagadougou. In the meantime, they also report changes in overwintering dates, an increase in extreme rainfall incidence and precipitation variability. There are several documented direct and indirect health impacts associated with such patterns such as increases in water-borne and vector borne diseases or food security ^10, 25, 26^. These patterns in regards to the change in precipitation regimes with increases in the frequency of extreme wet and dry years are known to be intensified in the context of climate change ^27^.

In parallel, some individuals might be escaping slow-onset disasters, such as rising sea levels and declining agricultural yields; their migration patterns may be more similar to those of rural–urban migrants, and they might experience many similar obstacles and barriers to their health as well ^28^. It can be observed from the literature that some health related challenges may be identical between these migrant groups: First, the re-emergence of infectious diseases and geographical migration of diseases ^29^. Migrants spatially re-distribute infections from endemic areas to new populations; they are also exposed to new diseases due to unsanitary living conditions. Second, reduced access to healthcare services: mass migration applies population pressure which can exceed the capacity of the local health and social services. Perceptions of long wait times, confusing administrative procedures, or discrimination also impede health system access for migrants ^30^. Third, disrupted social support networks contribute to adverse mental health outcomes ^31^, higher risk of violence, and spread of STIs, including HIV infection. Migrants are often perceived as potential security challenges for countries ^18, 32^. Niger is one example that has conducted research to understand the phenomenon of infectious diseases and migration, and how the health system can best adapt ( Box 2).

Box 2. **Malaria and migration in Niger**

Niger, and it’s Agadez region, has long been known as a crossroads for the regional transhumance and immigration to the North of the Country. Agadez is one of the driest regions of the country with a very low and irregular rainfall level and therefore it’s classified as a hypo-endemic region for malaria ^33^. In 2016, Agadez region reported 55411 malaria cases, 37% in adults aged 25 over and 20% in children aged from 1 to 4. These data contrast with the other countries where adults aged 25 and over account for only 17.4% and children aged 1 to 4 account for 42.6% of malaria cases ^34^. In fact, this is not an isolated case because the data for the last 6 years show a similar pattern. This may be explained in part by the irregularity of malaria transmission, which can lead to a loss of immunity to malaria by the population ^35^. However, it is also important to consider that people that travel through this region are primarily young adults. One hypothesis could be that several cases reported as indigenous cases are, in fact, exported cases that have very different profiles ( *Plasmodium falciparum* strain, drug resistance, associated pathology, behaviour toward the illness, etc.). Niger’s malaria control programs must adapt to these challenges.

However, the lack of consensus on what constitutes a climate change migrant suggests that the same concept is defined differently across a wide range of non-integrated disciplines, leading to poor documentation of the health needs and health seeking behavioral patterns of climate change migrants.

## Climate change and health systems

With its inclusion in Goal 3 of The Sustainable Development Agenda, the concept of Universal Health Coverage (UHC) has obtained consensus from the international community ^36^. UHC, regarded as the third global health transition ^37^, or, according to former WHO director Margaret Chan, “ *the most effective concept that public health can offer*”, aims at ensure access to good quality care and limit the impoverishment of people as a result of their illness ^38^. In September 2015, the Director of WHO/PAHO for the Americas, Carissa F. Etienne, stated that “ *we must all cooperate to reduce those factors that are contributing to climate change and to mitigate its health effects.”* Health systems are one of the major mediators in this relationship between climate change and population health. Consecutively, in September 2017, the new WHO Director-General has set UHC as his greatest challenge and highlighted at the UN General Assembly on Migration Health in New York City that “ *health systems must be sensitive to the needs of migrants.”* The direct and indirect effects of climate change on population health and disease development are now well discussed ^5, 39^, but there is still little literature on the health effects of migration (within and between countries) influenced by natural disasters and droughts exacerbated by climate change ^5^. In addition, the role of the health care system as a social determinant of health ^40^ and its capacity to protect populations affected by climate change was recently identified by WHO ^6^ and the Canadian Public Health Association (CPHA) ^41^. Following the famous Canadian approach to health promotion and the social determinants of health, CPHA emphasizes, for example, the principles and practices of environmentally responsible health care.

Health systems (and health professionals) suffer the shocks provoked by climate change and migration ^42, 43^. These shocks can be the direct consequence of climate change (floods, heat waves, hurricanes, etc) or indirect effects, i.e. the influx of patients suffering from diseases whose emergence or abnormally high frequency is due to climate change ^44^. Therefore, health care systems need to adapt to population migration (in and across countries) due to climate changes by considering the effects of both phenomena: 1), the diseases epidemiology evolution ^45^ (e.g. dengue vs malaria) and its impact for the population behavior and important skills for health professionals and 2) the identification and response to specific social (e.g. social acceptability of migrants) ^46^ and health problems of patients and professionals (e.g. mental health) in this context. In this sense, there is a very close link between UHC and emergency preparedness, as the WHO has just pointed out calling for “ *a mutual reinforcement of emergency preparedness and health systems strengthening strategies*”. Health security must also be achieved through good health systems preparedness for disasters caused by climate change ^47^. The capacity of health systems and their actors to prepare for and adapt to these climate-related shocks is known as *resilience*.

Current research practice largely overlooks the interconnection between climate change, migration, and health system, so the three areas of work are largely treated in isolation from one another. However, to better understand how health systems may be resilient to climate change shocks, the collaboration and integration of different areas of work is needed.

## Health systems resilience in the climate change context: still unclear concepts

According to the Sendai Framework (2015–2030) adopted at the Third United Nations World Conference on Disaster Risk Reduction in March 2015, it is essential “ *to enhance the resilience of national health systems*” ^48^. Still, very little attention has been paid to the role of the health system resilience in responding to climate change ^42, 43, 49^. One of the major global health journals ( *Health Policy and Planning*) released in November 2017 the first, to our knowledge, supplement issue about “Resilient and Responsive Health Systems” ^50^. None of the 11 articles, however, addressed climate change. Similarly, in 2015, WHO proposed an operational framework to build climate resilient health systems within the context of climate change ^42^, but the scientific and empirical basis for its production is unclear, and the issue of population migration is not mentioned.

Thus, the question of health system resilience regarding climate migration is still in its infancy regarding the concept itself and its indicators.

Resilience is a longstanding concept in the disciplines of life sciences, psychology ( Box 3) and climate change ^51^, but it is relative new to the study of health systems ^43, 52, 53^. Health system are compounded of both hardware (structure, organization, technology, resourcing) and software (values, norms, actors, relationships) components, and their resilience requires that they be understood and measured accordingly ^54^.

Box 3. **The origin of the concept of resilience in the field of psychology and its applicability on climate changes consequences today**

According to the Merriam Webster dictionary, the first use of the term resilience dates back to 1807. It was then used in physics about the ability of materials to resist shocks or regain their original shape after being compressed or deformed ^55^. During the 1970s, in community psychiatry, we look at the phenomenon of so-called "invulnerable" children who, in the confrontation of stress and adversity, do not develop psychological disorders. In 1979, the child psychiatrist Michael Rutter uses the term resilience to describe these children he is studying to understand what are the protective factors that allow them to cope with stress ^56, 57^. His work has notably helped to identify social support as one of the main protective factors. The definition of resilience used today to study the capacity of health systems to cope with the consequences of climate change is consistent with this work. The Intergovernmental Panel on Climate Change definbes resilience as: “ *the capacity of social, economic, and environmental systems to cope with a hazardous event or trend or disturbance, responding or reorganizing in ways that maintain their essential function, identity, and structure, while also maintaining the capacity for adaptation, learning, and transformation*” ^58^.

Recently, an article has developed a non-normative index for assessing the resilience of health systems, but its validation has not yet been completed ^59^. The Lancet Countdown paper series has adopted an iterative and open approach to the development of indicators to identify the links between climate change and public health. The 2018 Lancet Countdown report suggests some indicators in its section 2 to point out how the health sector should be at the forefront of adaptation efforts, ensuring health systems, hospitals, and clinics remain anchors of community resilience. Among those, indicators 2.1, 2.4, 2.6; 2.7, 2.8,) ( Box 4), can be useful to understand the link between climate change and health system resilience. Although the concept of health system resilience adoption is still limited and “ *does not capture the quality or effectiveness of efforts*”, as it was described for the 2017 report ^5, 60^ neither the resilience of health staff nor community is taken into account. The authors of this manuscript consider the selected indicators as a good example to highlight the still reductionist and uni-disicipline approach of how *resilience* is interpreted.

Box 4. **Some 2018 Lancet Countdown indicators about climate change and health systems ^60^**

Indicator 2.1: National adaptation plans for health

Indicator 2.4: Climate change adaptation to vulnerabilities from mosquito-borne diseases

Indicator 2.6: National assessments of climate change impacts, vulnerability, and adaptation for health

Indicator 2.7: Spending on adaptation for health and health-related activities

Indicator 2.8: Health adaptation funding from global climate financing mechanisms

Health systems’ resilience cannot be evaluated only in terms of infrastructures. In contrast, from a more holistic and fundamental research perspective, several recent articles propose conceptual frameworks ^52, 53, 59^ that suggest analysing the five main dimensions of a resilient system: awareness, diversity, self-regulation, integration, and adaptiveness ^53^.

## For interdisciplinary research

As described above with reference to existing literature, current research practice largely overlooks interconnections between climate change, migration, and health system. Typically, these 3 areas of work are treated by different groups of scholars, and the various dimensions of the links between migration and health are understood in isolation ^45^. In the same way, migration, climate, population’s health and resilience of health systems are typically analysed as separate components through disciplines and approaches in silos. Research on the intersection between all these components is very scarce. Consequently, there are gaps and a predominant compartmented analysis on the existing links between all of them. In contrast, interdisciplinary indicates a certain level of integration of knowledge, methods and/or ideas to construct and analyse the issue of study ^61, 62^. Hence, interdisciplinary research can lead to a better understanding of the links between migration and health. By applying mixed methods ^63^, and the collaboration of environmental, health and social sciences, strategies can be informed and interventions to protect population health. “ *By learning from other researchers one increases the possibilities of creative solutions*” ^64^.

Climate change is one of the main challenges of our century, having the potential to trigger important changes in population health which includes forcing migration. The role of health systems in the context of targeting universal health coverage may be central to address these challenges. Moreover, in the contexts of vulnerable populations and victims of climate change, health systems certainly have a very important role to play in preventing and alleviating health problems. However, vulnerable populations must be prepared to address these challenges and their resilience to climate change and potential subsequent population movements (climate migrants) is essential. This is why, for example, countries in the Americas Region adopted their health systems resilience policy in 2016 in favor of the UHC ^65^.

As revealed in this manuscript, the research on the intersection between climate change, health systems, and migrants is still very scarce. Because of its complexity, we need to move from a multidisciplinary (collaboration of different disciplines not necessarily from the beginning and towards a same issue) to an interdisciplinary approach (integration of different disciplines usually through a common design for a integration and holistic understanding of the same issue) ^64^ to understand the multiple pathways that link migration driven by climate change and population’s health.

Climate change, and in particular the issue of climate migration, is an extremely complex issue at the crossroads of multiple and fragmented research sectors (migration, population, health system, climate). The guide for interaction of the SDGs is a perfect illustration of the importance of this intersectorality ^66^. Thus, in the face of this complexity, it becomes impossible to mobilize fragmented disciplinary approaches in silos (earth science, demography, political science, economics, anthropology, clinical science, etc.) because they alone will not make it possible to understand the holistic nature of the phenomenon of the relationship between climate migration and health systems. This interdisciplinary approach, “which requires, rather than avoids, disciplinary specialization” ^64^ is also essential to understand the concept of health system resilience because knowledge about it is still too fragmented. A recent scoping review of the literature shows that the conceptual of health system resilience has not yet been sufficiently studied from an interdisciplinary perspective ^67^. As Bhaskar *et al.* (2017) described “by learning from other researchers one increases the possibilities of creatives solutions” ^64^ (4) and we definitely need solutions to improve the resilience of health systems for vulnerable population. As a very recent comprehensive review argues, further investments in interdisciplinarity collaborations should be made to unravel the link between climate change, migration, and health system resilience ^68^. It is therefore necessary to move beyond sectoral and disciplinary approaches to engage in intersectoral, systemic and interdisciplinary research programs.

**Figure 1.**
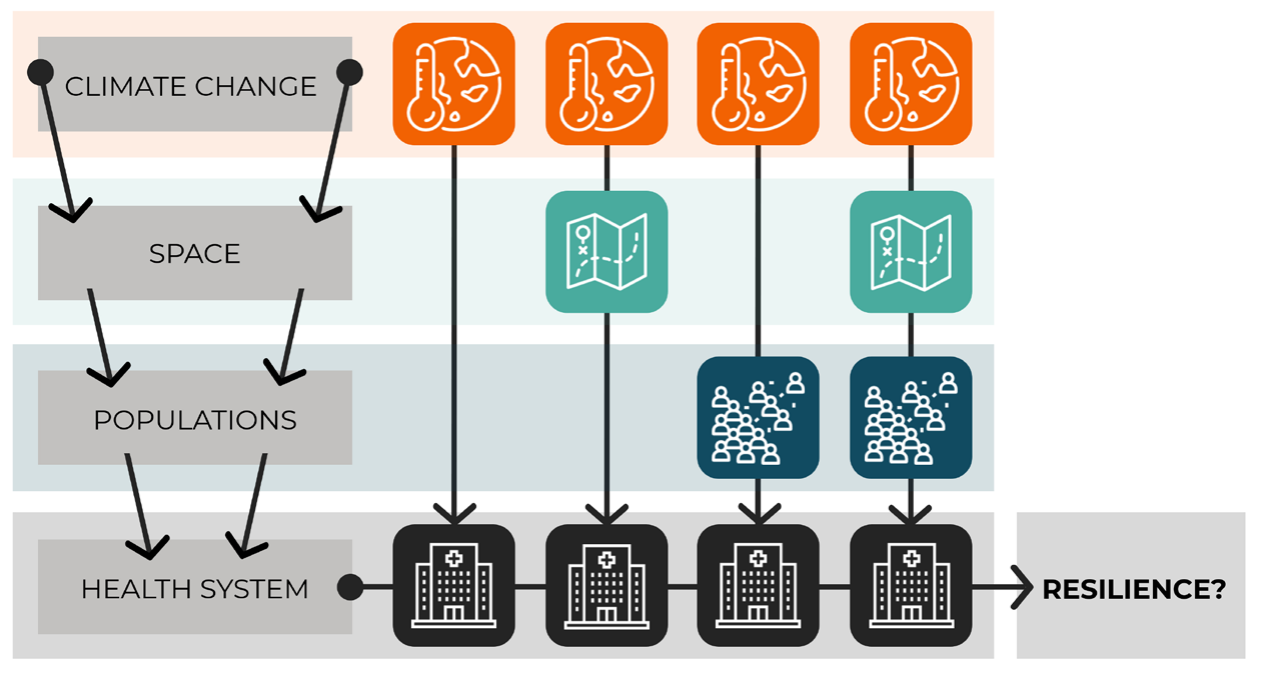
Health systems resilience in the climate change context.

We propose a series of interdisciplinary research questions to provide initial guidance in this direction ( Box 5). In Table 1 and Figure 1, we suggest a first summarization attempt of the challenges triggered by climate change for the resilience of health systems.

Box 5. **Some (non-exhaustive) future research questions**

- How is the concept of climate migrant delineated?
- What conceptual frameworks can support research on health systems’ resilience to climate change?
- In what ways are the health systems resilient to climate change-related migration?
- What role does climate change play in population movements and what are the health impacts?
- How do people displaced by climate change have access to health systems?
- How to promote health systems’ preparedness and resilience in the face of climate change?

**Table 1.** Pathways, scenarios and challenges between climate change, migrations and health systems resilience.

		Challenges for the health system resilience	
Pathways	Possible scenarios	Hard	Soft
1- Climate => Health System	Heat wave, extreme cold	Adaptation of buildings, targeted financing, electricity and water, cold chain strengthening, solar power, health staff uniforms	Engineer and health staff training, ability of the staff to work (and live) on extreme conditions
2- Climate => Space => Health System	Flood, hurricane	Adaptation and location of health facilities, emergency referral system, emergency preparedness	Disaster preparedness training for care and logistics (e.g. drugs), staff delay, staff moods and mental health
3- Climate => Local Population => Health System	Epidemics, new pathologies (dehydration, dengue, etc.),	Organization of an alert system, epidemiological surveillance, adaptation / forecasting of diagnostic capacities (i.e dengue vs malaria tests), vector control prevention	Staff training (pathologies, tests, differential diagnostic, etc.), relationships and trust with the population and between the staffs
4- Climate => Space => Displaced populations => Health System	Population movements, spread of (new) parasites / viruses, mental health	Logistics anticipation of patients' care, free healthcare, surveillance system, emergency referral system	Migration of staff, social acceptance of the arrival of displaced population and free care for them (all), training of health staff (languages, pathologies, etc.)

Figure 1 illustrates the different possible pathways, the details of which are presented in Table 1. We present them as exploratory to show how many hypotheses there are to test and how many research questions are open. It also shows how only an interdisciplinary approach can certainly help us to respond to them.

The first column of Table 1 proposes four different pathways involving the four elements that concern us here: climate, health system, space, and population. These pathways are more or less direct or complex as shown in Figure 1. The second column presents the possible scenarios in the context of each of these pathways and the last two columns present the challenges they pose to the resilience of health systems. For the first pathway (1), we believe that heat waves and extreme cold pose challenges to health systems (e.g. engineering). The second pathway (2) explains, for example, that climate change can cause floods or hurricanes, which impacts space (territory) and poses new challenges to the resilience of health systems (e.g. training health personnel in disaster preparedness). The third pathway (3) postulates that climate change will have direct effects on local populations, such as the presence of dengue fever in areas where malaria was endemic, which in turn will require the health system and its actors (e.g. power and trust issues) to adapt to these epidemic or pathological changes. Finally, the last pathway (4) we propose is at the heart of our discussion. We propose that it is essential to develop interdisciplinary research to better understand the effects of climate change causing spatial change events (e. g. floods) and thus forcing populations to migrate (within or between countries), which can have major effects on the resilience of health systems (in home or host countries).

Table 1 is proposed for illustrative purposes, but it shows the complexity of the phenomenon and the multitude of pathways that interdisciplinary research could explore.

## Data availability

No data are associated with this article.

## References

[1] Charlesworth E Ahmed I: Factors Pertaining to Building Resilience in Urban Slum Settlements of Dhaka, Bangladesh. Melbourne, VIC: Architects Without Frontiers (AWF);2012;18 Reference Source

[2] Verner D: Labor Markets in Rural and Urban Haiti Based on the First Household Survey for Haiti. World Bank. (POLICY RESEARCH WORKING PAPER 4574);2008;29 Reference Source

[3] Laczko F, editor: Migration, environment and climate change: assessing the evidence. Geneva: Internat. Organization for Migration;2009;441 Reference Source

[4] Warn E Adamo SB: The Impact of Climate Change: Migration and Cities in South America.World Meteorological Organization.2015; [cited 2017 Sep 7]. Reference Source

[5] Watts N Amann M Ayeb-Karlsson S: The *Lancet* Countdown on health and climate change: from 25 years of inaction to a global transformation for public health. *Lancet.* 2018;391(10120):581–630, [cited 2017 Nov 1]. 10.1016/S0140-6736(17)32464-9 29096948

[6] WHO: Protecting health from climate change connecting science, policy, and people. Copenhagen, Denmark: World Health Organization Regional Office for Europe;2009 Reference Source

[7] Papworth A Maslin M Randalls S: Is climate change the greatest threat to global health?: Commentary. *Geogr J.* 2015;181(4):413–22. 10.1111/geoj.12127

[8] Whitmee S Haines A Beyrer C: Safeguarding human health in the Anthropocene epoch: report of The Rockefeller Foundation- *Lancet* Commission on planetary health. *Lancet.* 2015;386(10007):1973–2028. 10.1016/S0140-6736(15)60901-1 26188744

[9] McLeman RA Hunter LM: Migration in the context of vulnerability and adaptation to climate change: insights from analogues. *Wiley Interdiscip Rev Clim Change.* 2010;1(3):450–61. 10.1002/wcc.51 22022342 PMC3183747

[10] Stanke C Kerac M Prudhomme C: Health effects of drought: a systematic review of the evidence. *PLoS Curr.* 2013;5: pii: ecurrents.dis.7a2cee9e980f91ad7697b570bcc4b004. 10.1371/currents.dis.7a2cee9e980f91ad7697b570bcc4b004 23787891 PMC3682759

[11] Mora C Spirandelli D Franklin EC: Broad threat to humanity from cumulative climate hazards intensified by greenhouse gas emissions. *Nat Clim Chang.* 2018;8(12):1062–71. 10.1038/s41558-018-0315-6

[12] Missirian A Schlenker W: Asylum applications respond to temperature fluctuations. *Science.* 2017;358(6370):1610–4. 10.1126/science.aao0432 29269476

[13] McMichael C Barnett J McMichael AJ: An ill wind? Climate change, migration, and health. *Environ Health Perspect.* 2012;120(5):646–54. 10.1289/ehp.1104375 22266739 PMC3346786

[14] Myers N: Environmental refugees: a growing phenomenon of the 21st century. *Philos Trans R Soc Lond B Biol Sci.* 2002;357(1420):609–13. 10.1098/rstb.2001.0953 12028796 PMC1692964

[15] Biermann F Boas I: Preparing for a Warmer World: Towards a Global Governance System to Protect Climate Refugees. *Glob Environ Polit.* 2010;10(1):60–88. 10.1162/glep.2010.10.1.60

[16] Migration, Environment and Climate Change: Assessing the Evidence - | IOM Online Bookstore.[cited 2017 Sep 15]. Reference Source

[17] Rechkemmer A O’Connor A Rai A: A complex social-ecological disaster: Environmentally induced forced migration. *Disaster Health.* 2016;3(4):112–20. 10.1080/21665044.2016.1263519 28265487 PMC5325461

[18] Brown O: Migration and Climate Change. Geneva: International Organisation for Migration;2008 (Research Series, No. 31). Reference Source

[19] Ahmed I: Building Resilience of Urban Slums in Dhaka, Bangladesh. *Procedia Soc Behav Sci.* 2016;218:202–13. 10.1016/j.sbspro.2016.04.023

[20] Kreft S Eckstein D Dorsch L: Global climate risk index 2016: Who Suffers Most From Extreme Weather Events? Weather-related Loss Events in 2014 and 1995 to 2014.Berlin: Germanwatch e.V;2016;32 Reference Source

[21] WHO: The World Health Report 2000 - Health Systems: Improving Performance.2000 Reference Source

[22] Kreft S Eckstein D Dorsch L: Global climate risk index 2016: Who Suffers Most From Extreme Weather Events? Weather-related Loss Events in 2014 and 1995 to 2014. Berlin: Germanwatch e.V.2017;32 Reference Source

[23] Heaney AK Winter SJ: Climate-driven migration: an exploratory case study of Maasai health perceptions and help-seeking behaviors. *Int J Public Health.* 2016;61(6):641–9. 10.1007/s00038-015-0759-7 26552667

[24] Bonnet E Amalric M Nikiema A: Connaissances des inondations par les ouagalais.Ouagadougou, Burkina Faso: IRD.2017;4 Reference Source

[25] Sena A Ebi KL Freitas C: Indicators to measure risk of disaster associated with drought: Implications for the health sector.Zia A, editor. *PLoS One.* 2017;12(7):e0181394. 10.1371/journal.pone.0181394 28742848 PMC5526563

[26] Levy K Woster AP Goldstein RS: Untangling the Impacts of Climate Change on Waterborne Diseases: a Systematic Review of Relationships between Diarrheal Diseases and Temperature, Rainfall, Flooding, and Drought. *Environ Sci Technol.* 2016;50(10):4905–22. 10.1021/acs.est.5b06186 27058059 PMC5468171

[27] Polade SD Gershunov A Cayan DR: Precipitation in a warming world: Assessing projected hydro-climate changes in California and other Mediterranean climate regions. *Sci Rep.* 2017;7(1):10783. [cited 2017 Dec 17]. 10.1038/s41598-017-11285-y 28883636 PMC5589768

[28] McMichael C: Climate change-related migration and infectious disease. *Virulence.* 2015;6(6):548–53. 10.1080/21505594.2015.1021539 26151221 PMC4720222

[29] Chase LE Cleveland J Beatson J: The gap between entitlement and access to healthcare: An analysis of "candidacy" in the help-seeking trajectories of asylum seekers in Montreal. *Soc Sci Med.* 2017;182:52–9. 10.1016/j.socscimed.2017.03.038 28412641

[30] Torres JM Casey JA: The centrality of social ties to climate migration and mental health. *BMC Public Health.* 2017;17(1):600. 10.1186/s12889-017-4508-0 28679398 PMC5498922

[31] Marcelin LH Cela T Shultz JM: Haiti and the politics of governance and community responses to Hurricane Matthew. *Disaster Health.* 2016;3(4):151–61. 10.1080/21665044.2016.1263539 28321361 PMC5351817

[32] Petkova EP Ebi KL Culp D: Climate Change and Health on the U.S. Gulf Coast: Public Health Adaptation is Needed to Address Future Risks. *Int J Environ Res Public Health.* 2015;12(8):9342–56. 10.3390/ijerph120809342 26270669 PMC4555284

[33] Julvez J Develoux M Mounkaila A: [Diversity of malaria in the Sahelo-Saharan region. A review apropos of the status in Niger, West Africa]. *Ann Soc Belg Med Trop.* 1992;72(3):163–77. 1476465

[34] Direction des Statistiques du Ministère de la Santé Publique: Annuaire des statistiques sanitaires du Niger année 2016.2017 Reference Source

[35] Doudou MH Mahamadou A Ouba I: A refined estimate of the malaria burden in Niger. *Malar J.* 2012;11(1):89. [cited 2017 Nov 14]. 10.1186/1475-2875-11-89 22453027 PMC3342108

[36] Robert E Lemoine A Ridde V: Que cache le consensus des acteurs de la santé mondiale au sujet de la couverture sanitaire universelle? Une analyse fondée sur l’approche par les droits. *Can J Dev Stud.* 2017;38(2):199–215. 10.1080/02255189.2017.1301250

[37] Rodin J de Ferranti D: Universal health coverage: the third global health transition? *Lancet.* 2012;380(9845):861–2. 10.1016/S0140-6736(12)61340-3 22959371

[38] WHO: Arguing for universal health coverage. Geneva: World Health Organization.2013;39 Reference Source

[39] Watts N Adger WN Agnolucci P: Health and climate change: policy responses to protect public health. *Lancet.* 2015;386(10006):1861–914. 10.1016/S0140-6736(15)60854-6 26111439

[40] Evans RG Barer ML Marmor TR: Why are some people healthy and others not?: the determinants of health of populations.New York: A. de Gruyter; (Social institutions and social change).1994; **xix**:378 Reference Source

[41] CPHA: Global Change and Public Health: Addressing the Ecological Determinants of Health.Ottawa: Canadian Public Health Association Discussion Document.2015;36 Reference Source

[42] WHO: Operational framework for building climate resilient health systems. Switzerland: World Health Organization.2015;47 Reference Source

[43] Witter S Hunter B: Resilience of health systems during and after crises – what does it mean and how can it be enhanced?London: ReBUILD Consortium.2017;4 Reference Source

[44] Verner G Schütte S Knop J: Health in climate change research from 1990 to 2014: positive trend, but still underperforming. *Glob Health Action.* 2016;9(1): 30723. 10.3402/gha.v9.30723 27339855 PMC4917601

[45] Pan W: Migration as a mediator of climate-related infectious disease risk [Internet].2018;6 Reference Source

[46] Witter S Wurie H Chandiwana P: How do health workers experience and cope with shocks? Learning from four fragile and conflict-affected health systems in Uganda, Sierra Leone, Zimbabwe and Cambodia. *Health Policy Plan.* 2017;32(Suppl_3):iii3–13. 10.1093/heapol/czx112 29149313

[47] Schmets G Hanssen O Soucat A: Interconnectedness of UHC and health security.Background paper developed for the 9th Global Meeting of Heads of WHO offices. Geneva, Switzerland: WHO.2017;5 Reference Source

[48] UNISRD: Sendai Framework for Disaster Risk Reduction 2015–2030. Geneva, Switzerland: UNISRD.2015;38 Reference Source

[49] Ridde V Ramel P: The migrant crisis and health systems: Hygeia instead of Panacea. *Lancet Public Health.* 2017;2(10):e447. 10.1016/S2468-2667(17)30180-9 29253426

[50] Mills A: Resilient and responsive health systems in a changing world. *Health Policy Plan.* 2017;32(suppl_3):iii1–2. 10.1093/heapol/czx117 29149318

[51] Tanner T Bahadur A Moench M: Challenges for resilience policy and practice. London: Overseas Development Institute.2017;25 Reference Source

[52] Gilson L Barasa E Nxumalo N: Everyday resilience in district health systems: emerging insights from the front lines in Kenya and South Africa. *BMJ Glob Health.* 2017;2(2):e000224. 10.1136/bmjgh-2016-000224 29081995 PMC5656138

[53] Kruk ME Myers M Varpilah ST: What is a resilient health system? Lessons from Ebola. *Lancet.* 2015;385(9980):1910–2. 10.1016/S0140-6736(15)60755-3 25987159

[54] Gilson L, editor: Systems research. A methodology reader.Alliance for Health Policy and Systems Research. World Health Organization.2012;472 Reference Source

[55] Reid R Botterill LC: The Multiple Meanings of ‘Resilience’: An Overview of the Literature. *Aust J Publ Admin.* 2013;72(1):31–40. 10.1111/1467-8500.12009

[56] Rutter M: Resilience in the face of adversity. Protective factors and resistance to psychiatric disorder. *Br J Psychiatry.* 1985;147(6):598–611. 10.1192/bjp.147.6.598 3830321

[57] Rutter M: Protective factors in children’s responses to stress and disadvantage.In: *Primary prevention in psychopathology: Social competence in children* Hanover. University Press of New England.1979; **8**:49–74. 547874

[58] IPCC: Annex II: Glossary. In: *Climate Change 2014: Impacts, Adaptation, and Vulnerability Part B: Regional Aspects Contribution of Working Group II to the Fifth Assessment Report of the Intergovernmental Panel on Climate Change* Cambridge University Press.2014;1757–76. Reference Source

[59] Kruk ME Ling EJ Bitton A: Building resilient health systems: a proposal for a resilience index. *BMJ.* 2017;357:j2323. 10.1136/bmj.j2323 28536191

[60] Watts N Amann M Arnell N: The 2018 report of the *Lancet* Countdown on health and climate change: shaping the health of nations for centuries to come. *Lancet.* 2018;392(10163):2479–2514. 10.1016/S0140-6736(18)32594-7 30503045 PMC7616804

[61] Cloos P: Racialization, Between Power and Knowledge: A Postcolonial Reading of Public Health as a Discursive Practice1. *Journal of Critical Race Inquiry.* 2011;1(2):57–76. Reference Source

[62] Robert E Ridde V: Quatre principes de recherche pour comprendre les défis des systèmes de santé des pays à faible et moyen revenu. *Can J Public Health.* 2016;107(4–5):e362–e365. 10.17269/cjph.107.5533 31820357 PMC6972251

[63] Pluye P Hong QN: Combining the power of stories and the power of numbers: mixed methods research and mixed studies reviews. *Annu Rev Public Health.* 2014;35:29–45. 10.1146/annurev-publhealth-032013-182440 24188053

[64] Bhaskar R Danermark B Price L: Interdisciplinarity and wellbeing: a critical realist general theory of interdisciplinarity. Abingdon, Oxon; New York, NY: Routledge; (Routledge studies in critical realism).2017 10.4324/9781315177298

[65] PAHO: Resilience health system. [Internet] Washington: PAHO; (CD55/9).2016 Reference Source

[66] International Concil for Science: A Guide to SDG Interactions: from Science to Implementation.2017;237 Reference Source

[67] Paillard Turenne C Gautier L Degroote S: Conceptual analysis of health systems resilience: a scoping review. *Soc Sci Med.*In review. 10.1016/j.socscimed.2019.04.020 31100697

[68] Butler C: Climate Change, Health and Existential Risks to Civilization: A Comprehensive Review (1989–2013). *Int J Environ Res Public Health.* 2018;15(10): pii: E2266. 10.3390/ijerph15102266 30332777 PMC6210172

