# Supplementary material for: Climate change, migration and health systems resilience: Need for interdisciplinary research
Source: F1000Res. 2019 Apr 1;8:22. Originally published 2019 Jan 7. [Version 2] doi: 10.12688/f1000research.17559.2 (PMC7506192; doi:10.12688/f1000research.17559.2)
Supplement: Peer review history

# Peer review: Reviewer response for version 2

**DOI:** 10.5256/f1000research.20500.r70003

**Published:** 2020-10-16

**Authors:** Paddy Enright

**Affiliations:** 1 University of Waterloo, Waterloo, ON, Canada

Thank you for the opportunity to review this paper. It discusses a topic of growing importance, and hopefully equally growing interest within the research community. Health systems require adaptation in order to meet the needs of migrants and other vulnerable groups. As you point out, interdisciplinary research will be needed to develop, prioritize, implement, evaluate and refine the adaptations needed to build climate-resilient health systems.

 In summary your article discusses:

- The role of climate change in driving migration;
- The health impacts of climate change,
- The consequences of both climate change and climate-driven migration for health systems;
- How health system resilience can help address these issues; and
- The need for interdisciplinary research to help build climate-resilient health systems.

I think your paper, particularly Box 5, identifies many important areas where further investigation is needed in order to protect health and wellbeing in both the short and long-term. However, I believe there are some areas where the paper could be strengthened.

 In hopes of supporting your efforts to further strengthen the paper, I put forward the following comments for your consideration:

**Substantive Comments:**

- The abstract states that no research on climate change and health system resilience has taken place, I believe this to be incorrect (e.g. WHO Operational Framework for Building Climate Resilient Health Systems was published in 2015).
- The stated aim of the paper is to advance the case for interdisciplinary research. However, existing barriers to interdisciplinary research and potential options for overcoming these barriers are not discussed. Highlighting these barriers and suggesting options to address them would substantially add to the paper’s value.

- Though you state clearly that this is a non-systematic review, I am believe your search strategy limited your findings and the subsequent results of your paper. For example, by replacing the search term “migrants” with “migration” substantially more results are retrieved. I believe the search could have been further refined to help support the development of the paper. Additionally, a broader review of the grey literature (e.g. climate change and health vulnerability and adaptation assessments conducted by organizations such as the WHO and World Bank) may be beneficial.  

- The argumentation throughout the paper is loose and statements that appear definitive or conclusive are made without providing the evidence or context needed to support them. For example, readers are directed towards the text boxes for further information but the information in question isn’t concretely discussed within the text boxes.  
  - Consider revising Box 1 to link more directly with the text. Currently the text provides more insight on climate change perceptions than on the role of climate change in migration and health.
  - Currently Box 2 does not appear to discussion adaptation, as mentioned in the text that proceeds it (though it mentions adaptation is needed). Consider revising to either discuss adaptation or more concretely discuss the impacts of the intersection of migration and malaria on Niger’s health system(s).

- UHC is often discussed, but its role in building climate-resilient health systems isn’t made clear. Consider revising the text to better make the connection between UHC and climate-resilient health systems. Additionally, UHC systems still require adaptation to be climate-resilient, what specific adaptations would most benefit migrants?

- The text describing the vulnerability of health systems to climate change could be stronger. I suggest clearly defining what is meant by a health system at the beginning of this section (paragraph 1 page 5 of the pdf version) and detailing examples of impacts to each constituent component (e.g. infrastructure, health workforce, health information systems, health policy and governance, etc.).  Providing additional context on how these impacts will influence the health and wellbeing of migrants, or how migration may further strain the components of health systems, would also help readers make the link between health systems and migration and the need for interdisciplinary work in this area.

- You introduce the concept of resilience indicators and provide examples of efforts to measure resilience. However, you do not connect measuring climate-resilience to migration or make the case for resilience indicators that meet the needs of migrants and other vulnerable groups. I suggest revising this section to make the link to the health and wellbeing needs of migrants.

- Greater explanation is needed for how Figure 1 and Table 1 were developed. For example, I assume the possible scenarios column is not meant to be exhaustive? If not, consider making this clearer.

- In Table 1 additional explanation is required for the differentiation between hard and soft challenges and how this differentiation was made. For example, why is logistics a soft challenge? I would propose that the climate hazards that impact health systems may also disrupt health supply chains.

 

**Additional non-substantive items for your consideration:**

- In the development of the paper generally, but specifically Figure 1 and Table 1, was any consideration given to cascading or simultaneous hazards or disasters and their impacts? If not, considering the role of cascading hazards and their implications for migration and health systems may be a worthwhile area for future research.

- Given that you address the role of ‘space’ in Figure 1 and Table 1, has any consideration been given for the role of ‘place’? How do perceptions of place and identity impact the implications of migration from a health and health systems perspective? 

- Additional research items for consideration within Box 5:
  - How do the health impacts of climate change and access to health care differ for internally displaced populations versus international migrants?
  - What considerations are there for displaced Indigenous Peoples?
  - How is mental health and access to mental health care impacted by climate-driven migration? Do climate-driven changes to space and place impact the views of migrants on returning to their homes?
  - What added considerations are there for those displaced from rural and/or remote regions to urban centres?
  - What long-term implications are there for health systems planning and investments (particularly considering the long lifespan of many health system components)?

Is the topic of the opinion article discussed accurately in the context of the current literature?

Partly

Are arguments sufficiently supported by evidence from the published literature?

Partly

Are all factual statements correct and adequately supported by citations?

Partly

Are the conclusions drawn balanced and justified on the basis of the presented arguments?

Yes

Reviewer Expertise:

health impacts of climate change; climate-resilient health systems; health adaptation

I confirm that I have read this submission and believe that I have an appropriate level of expertise to confirm that it is of an acceptable scientific standard, however I have significant reservations, as outlined above.

# Peer review: Reviewer response for version 2

**DOI:** 10.5256/f1000research.20500.r70836

**Published:** 2020-09-21

**Authors:** Cunrui Huang

**Affiliations:** 1 School of Public Health, Sun Yat-sen University, Guangzhou, China

The article describes the significant future research directions on climate change, migration and health systems resilience, and stresses the importance of interdisciplinary methods. It is a topic of interest to the researchers in the related areas, but the article needs significant improvement.

 My detailed comments are as follows:

1. The article does not demonstrate the association between climate change, migration, and health system resilience well, making the full text seem rather fragmented. Table 1 and Figure 1 at the end of the article show the relationship and conceptual pathways among climate change, migration, and health system resilience. It might be more suitable to be discussed in detail in the previous part of the article instead of "For interdisciplinary research" section.
2. The article wants to highlight the importance of interdisciplinary research, but it is not well discussed in the "Climate migrants and health challenges" section.
3. In the “Climate migrants and health challenges” section, the authors use the example in Box 1 to illustrate that some climate immigrants do not realize how climate change influences and has an impact on their health needs and social patterns. However, this has nothing to do with the content before and after the article, nor can it intuitively reflect health challenges.
4. At the beginning of the “Climate change and health systems” section, the association between Universal Health Coverage and health systems is not well explained, making the introduction of Universal Health Coverage incongruent here.

Is the topic of the opinion article discussed accurately in the context of the current literature?

Yes

Are arguments sufficiently supported by evidence from the published literature?

Partly

Are all factual statements correct and adequately supported by citations?

Yes

Are the conclusions drawn balanced and justified on the basis of the presented arguments?

Yes

Reviewer Expertise:

Climate Change and health

I confirm that I have read this submission and believe that I have an appropriate level of expertise to confirm that it is of an acceptable scientific standard, however I have significant reservations, as outlined above.

# Peer review: Reviewer response for version 2

**DOI:** 10.5256/f1000research.20500.r46553

**Published:** 2019-06-24

**Authors:** Lucy Gilson

**Affiliations:** 1 Health Policy and Systems Division, School of Public Health and Family Medicine, University of Cape Town, Cape Town, South Africa

Thanks to the authors for these revisions, which strengthen the paper.

Is the topic of the opinion article discussed accurately in the context of the current literature?

Yes

Are arguments sufficiently supported by evidence from the published literature?

Partly

Are all factual statements correct and adequately supported by citations?

Yes

Are the conclusions drawn balanced and justified on the basis of the presented arguments?

Partly

Reviewer Expertise:

Health policy and systems research

I confirm that I have read this submission and believe that I have an appropriate level of expertise to confirm that it is of an acceptable scientific standard.

# Peer review: Reviewer response for version 1

**DOI:** 10.5256/f1000research.19202.r42728

**Published:** 2019-02-11

**Authors:** Katharina Waha

**Affiliations:** 1 CSIRO Agriculture and Food (Commonwealth Scientific and Industrial Research Organisation), Brisbane, Queensland, Australia

The article calls for further research on climate change, migration and health system resilience and an interdisciplinary approach but does not present a convincing line of reasoning. Often the individual paragraphs seem unconnected.

 For example, in the section on “Climate migrants and health systems” I am not sure what the authors are trying to do or say. It’s a loose collection of thoughts to me at the moment. The first two paragraphs are about general impacts of climate change and migration and the authors are careful to not link them directly which is good. The next paragraph is about perception of migrants which is interesting and then the authors move into the Burkina Faso example where it is not clear if people have migrated at all.

 I think the topic is interesting and very relevant and one thing that might help to structure the article better, is to clarify whether the authors are interested in the effects of climate change on an *individual’s* health (migrants) or a *country’s* health system or both. This seems to be mixed up in the article. Table 1 and Figure 1 seems to sort of help with that, but they are not integrated in the text at all, they are just added at the end but should be central to the paper.

 Other comments:

- “The estimation that is most widely accepted is that over 200 million persons will be displaced globally by 2050 because of climate change ^13,15,18^”. Inappropriate use of literature. McMichael *et al.* (2012) (13) actually says that 200 million is the figure most widely accepted and refers to Myers (2002) as the source of that figure, but also says that the empirical basis has been questioned. This is an important consideration that needs to be added here. The other two references are not needed then, except if they are given to support the notion of ‘most widely accepted’ in which case I would expect more studies.
- Box 1: Conclusion in the last sentence about perceptions of local populations needing to be enhanced does not follow from previous paragraphs. The authors would have to establish a disconnect between perceptions of climate change and flooding and results from a detection and attribution study in order to conclude that.
- Box 4 and the resilience section: These indicators seem to be for resilience and adaptation planning, not just for resilience. The concept of resilience seems to be important in the article but only got mentioned once in the second last section and there it gets mixed up with adaptation indicators. The Lancet Countdown Report gives some of them as "Adaptation Planning and Resilience for Health Indicators". Can you strengthen this part and explain better why this is an important consideration?
- “The role of health systems in the context of targeting universal health coverage may be central to address these challenges.” The authors speak about universal health coverage only once before and do not give any reason for this conclusion.
- How are health needs and health system resilience different between “climate change migrants” and other migrants that e.g. flee war? The authors state that they “face challenges similar to those of refugees fleeing war and/or political persecution” and “might experience many similar obstacles and barriers to their health as well”, so why the need to study this topic separately?

Is the topic of the opinion article discussed accurately in the context of the current literature?

Partly

Are arguments sufficiently supported by evidence from the published literature?

Partly

Are all factual statements correct and adequately supported by citations?

Partly

Are the conclusions drawn balanced and justified on the basis of the presented arguments?

No

Reviewer Expertise:

Climate change impact research

I confirm that I have read this submission and believe that I have an appropriate level of expertise to confirm that it is of an acceptable scientific standard, however I have significant reservations, as outlined above.

# Response

**Published:** 2019-04-17

**Authors:** Ariadna Nebot

**Affiliations:** Independent Consultant, Pharmacist and Public Health Specialist, France

**Answers to Katharina Waha who approved with reservations**

- *The article calls for further research on climate change, migration and health system resilience and an interdisciplinary approach but does not present a convincing line of reasoning. Often the individual paragraphs seem unconnected.*

**Thank you for your feedback. In light of Lucy Gilson's comments, we have tried to reorganize some sections of the article. We hope that this will make more sense.**

 

- *For example, in the section on “Climate migrants and health systems” I am not sure what the authors are trying to do or say. It’s a loose collection of thoughts to me at the moment. The first two paragraphs are about general impacts of climate change and migration and the authors are careful to not link them directly which is good. The next paragraph is about perception of migrants which is interesting and then the authors move into the Burkina Faso example where it is not clear if people have migrated at all.*

**We have renamed this first section to better show that its objective is to set the scene for the relationship between climate migrants and health. We have made it clear that the people from Burkina Faso affected by Box 1 have been displaced by flooding, thank you for the comment.**

- * I think the topic is interesting and very relevant and one thing that might help to structure the article better, is to clarify whether the authors are interested in the effects of climate change on an individual’s health (migrants) or a country’s health system or both. This seems to be mixed up in the article. Table 1 and Figure 1 seems to sort of help with that, but they are not integrated in the text at all, they are just added at the end but should be central to the paper.* 

**We have tried to restructure the article to better show that what interests us is not so much the link between climate migrants and health but rather the link between climate migrants and health system because it has not yet been much addressed by research. We have added text to better integrate and explain Table 1 and Figure 1.**

 

- *Other comments:“The estimation that is most widely accepted is that over 200 million persons will be displaced globally by 2050 because of climate change13,15,18”. Inappropriate use of literature. McMichael et al. (2012) (13) actually says that 200 million is the figure most widely accepted and refers to Myers (2002) as the source of that figure, but also says that the empirical basis has been questioned. This is an important consideration that needs to be added here. The other two references are not needed then, except if they are given to support the notion of ‘most widely accepted’ in which case I would expect more studies.*

**Thanks for this very relevant comment. Accordingly, and in order to avoid confusion, we have replaced the figure with empirical basis questioned by the following sentence: “the simple fact is that nobody really knows with any certainty what climate change will mean for human population distribution. Current estimates range between 25 million and 1 billion people by 2050.” (Brown 2008) and we have also deleted the 2 references (15, 18); not necessary in supporting the argument anymore.**

 

- *Box 1: Conclusion in the last sentence about perceptions of local populations needing to be enhanced does not follow from previous paragraphs. The authors would have to establish a disconnect between perceptions of climate change and flooding and results from a detection and attribution study in order to conclude that.*

**Yes, thanks for this suggestion. We have just deleted the last sentence in order to clarify the disconnection suggested by the reviewer.**

 

- *Box 4 and the resilience section: These indicators seem to be for resilience and adaptation planning, not just for resilience. The concept of resilience seems to be important in the article but only got mentioned once in the second last section and there it gets mixed up with adaptation indicators. The Lancet Countdown Report gives some of them as "Adaptation Planning and Resilience for Health Indicators". Can you strengthen this part and explain better why this is an important consideration?*

**Yes, thanks for this comment. It is correct that the Lancet Countdown indicator’s section 2 refers to “adaptation, planning and resilience for health” and not only to resilience. The reason is of course that *adaptation *and *resilience* are directly related and this section 2 aims to put at the front the adaptation efforts to promote and achieve community resilience, as we can see in p.13 of the 2018 Lancet Countdown’s report:**

***“With the observed and future health impacts of climate change becoming increasingly evident, and emission trajectories committing the world to further warming, accelerated adaptation interventions are needed to safeguard populations’ health. As the 2030 agenda shows, 45 strategies to improve community resilience are often linked to poverty reduction and broader socioeconomic development imperatives, creating the possibility of no regret scenarios”.*** 

**However, in this same p.13, it is said that, although the 2018 Lancet Countdown report counts on improved indicators for this section, the community resilience is still few explored and that collected data give more insights in adaptation than in resilience:**

***“The health sector should be at the forefront of adaptation efforts, ensuring health systems, hospitals, and clinics remain anchors of community resilience. This  underrecognised, yet growing area of practice, is the focus of this section.”***

***The data are incomplete, providing more insight into adaptation than resilience, and predominantly allow for process-based indicators.”***

**Therefore, the authors of this paper considered some of these Lancet Countdown indicators as a good example to visibilise the still reductionist and uni-disicipline approach of how *resilience* is interpreted In order to make this intention more explicit; we have added these two-lines in p 7.**

 

- *“The role of health systems in the context of targeting universal health coverage may be central to address these challenges.” The authors speak about universal health coverage only once before and do not give any reason for this conclusion.*

**We have added some clarifications to this sentence, p 9**.

 

- *How are health needs and health system resilience different between “climate change migrants” and other migrants that e.g. flee war? The authors state that they “face challenges similar to those of refugees fleeing war and/or political persecution” and “might experience many similar obstacles and barriers to their health as well”, so why the need to study this topic separately?*

 

**Thanks for this comment. According to what we could find in the literature (and therefore, what is already documented) climate migrants health needs may share similar patterns to refugees and/or to rural-urban migrants (P.4). However, in this same paragraph, we also mention the additional vulnerability that this category of population may have: “ *In addition, environmental change migrant population is usually the most vulnerable as well because migration is often expensive and climate change factors can easily lie on the top of other strong socio-economic factor.”***

**Considering this ‘additional vulnerability’, the author’s underlying hypothesis may be that climate migrants health needs and health system resilience may be slightly different. However, the non-integrated disciplines that can be looking at that doesn’t allow to further explore this specificities. We have modified the last statement of this paragraph in order to strengthen this idea *: “However, the lack of consensus of climate change migrant suggests how the same phenomenon is defined from different and non-integrated disciplines and, therefore,  how climate change migrant health needs and patterns are still scarcely documented. “***

# Peer review: Reviewer response for version 1

**DOI:** 10.5256/f1000research.19202.r42732

**Published:** 2019-02-04

**Authors:** Lucy Gilson

**Affiliations:** 1 Health Policy and Systems Division, School of Public Health and Family Medicine, University of Cape Town, Cape Town, South Africa

This is an important paper on a vital topic. It provides useful directions for future research around climate change, migration and health system resilience. Nonetheless, the overall argumentation of the paper is not fully clear – and so it is difficult fully to judge the use of evidence and assess the conclusions.

 The broad argument seems to be intended as:

1. Climate change and migration are inter-linked and have negative health consequences (‘climate migrants and health systems’).
2. Health systems are vital to tackling public health challenges such as those of climate change and migration (‘climate change in the global health context’).
3. Whilst there is increasing focus on health system resilience, this has not yet included concern for climate change or migration (‘health systems resilience in the climate change context’).
4. There is a need for ‘interdisciplinary research’ on climate change, health systems and migrants (‘for interdisciplinary research’). 

However, whilst the last section presents a case for interdisciplinary research, the earlier sections essentially work towards the conclusion that climate change, migration and health systems are interlinked. In addition, although there is reference to the point that current research is conducted in silos with little consideration of the intersection between these terrains on p.5, this point is not clearly argued previously in the paper.

 In supporting the final step of the paper’s argument (point 4 above), I suggest, then, that there would be value in strengthening the argument around the current silo-based nature of research in these domains as well as discussing further why and how interdisciplinary research is valuable for this area of work. I propose placing both these sets of issues in the section ‘for interdisciplinary research’ (some are currently in the previous section). There may also be value in clarifying that in this context ‘disciplines’ are, I think, equated to areas of work (climate change, migration, health systems) as opposed to e.g. sociology, anthropology, clinical science etc. And then I suggest it would be helpful to: expand on the point that ‘interdisciplinary’ means ‘a certain level of integration of knowledge, methods and/or ideas’ (p.5), and to discuss more than the need for mixed methods; clarify why an interdisciplinary approach is better than a multidisciplinary one for this work (p.5); and deepen the point about the value of the focus on resilience and adaptive strategies in supporting interdisciplinary research (p.5) – as well as explaining more of the detail of Table 1 and Figure 1. (As an aside, in Table 1 I would propose there would be value in thinking about health system software as more than staff training, essentially; relationships among staff within the system and with the public also matter, for example).

 In terms of the earlier sections of the paper I was not sure why the first section is titled ‘climate migrants and health systems’, as the focus is on health challenges rather than health systems. I also found that the logic and structure of the sections ‘climate change in the global health context’, and ‘health systems resilience in the climate change context’ made it difficult to follow the argument within them. In ‘climate change in the global health context’, this might be because the very tight referencing practice has overshadowed the argument. In ‘health systems resilience in the climate change context’, the linkage between the different points presented is not very clear (i.e. the argument connecting them).

 Some other minor points for review in p.3:

- What is an heuristic literature review?
- How are the 10 papers that were identified in the PubMed search used in the text, or is the point here that *only *10 papers were identified?
- At the first mention, briefly clarify the significance of the Lancet Countdown for this paper.

One final comment: given that this is a very closely argued piece, there would be value in some really close copy editing – as, for example, missing words in sentences, long sentences, and sentences that are phrased quite clumsily, hinder understanding.

Is the topic of the opinion article discussed accurately in the context of the current literature?

Yes

Are arguments sufficiently supported by evidence from the published literature?

Partly

Are all factual statements correct and adequately supported by citations?

Yes

Are the conclusions drawn balanced and justified on the basis of the presented arguments?

Partly

Reviewer Expertise:

Health policy and systems research

I confirm that I have read this submission and believe that I have an appropriate level of expertise to confirm that it is of an acceptable scientific standard, however I have significant reservations, as outlined above.

# Response

**Published:** 2019-04-17

**Authors:** Ariadna Nebot

**Affiliations:** Independent Consultant, Pharmacist and Public Health Specialist, France

**Answers to Lucy Gilson's comments, who approved with reservations**

- *This is an important paper on a vital topic. It provides useful directions for future research around climate change, migration and health system resilience. Nonetheless, the overall argumentation of the paper is not fully clear – and so it is difficult fully to judge the use of evidence and assess the conclusions.*
- *The broad argument seems to be intended as:* *Climate change and migration are inter-linked and have negative health consequences (‘climate migrants and health systems’).* *Health systems are vital to tackling public health challenges such as those of climate change and migration (‘climate change in the global health context’).* *Whilst there is increasing focus on health system resilience, this has not yet included concern for climate change or migration (‘health systems resilience in the climate change context’).* *There is a need for ‘interdisciplinary research’ on climate change, health systems and migrants (‘for interdisciplinary research’).*

**Thank you for this summary which indeed corresponds to the approach we adopted in our paper**.

 

- *However, whilst the last section presents a case for interdisciplinary research, the earlier sections essentially work towards the conclusion that climate change, migration and health systems are interlinked. In addition, although there is reference to the point that current research is conducted in silos with little consideration of the intersection between these terrains on p.5, this point is not clearly argued previously in the paper.*
- *In supporting the final step of the paper’s argument (point 4 above), I suggest, then, that there would be value in strengthening the argument around the current silo-based nature of research in these domains as well as discussing further why and how interdisciplinary research is valuable for this area of work. I propose placing both these sets of issues in the section ‘for interdisciplinary research’ (some are currently in the previous section). There may also be value in clarifying that in this context ‘disciplines’ are, I think, equated to areas of work (climate change, migration, health systems) as opposed to e.g. sociology, anthropology, clinical science etc. And then I suggest it would be helpful to: expand on the point that ‘interdisciplinary’ means ‘a certain level of integration of knowledge, methods and/or ideas’ (p.5), and to discuss more than the need for mixed methods; clarify why an interdisciplinary approach is better than a multidisciplinary one for this work (p.5); and deepen the point about the value of the focus on resilience and adaptive strategies in supporting interdisciplinary research (p.5)*

**Thanks for this very pertinent comment. Following your recommendations, we have reviewed each section and introduced at the end of section 1, section 2 and section 4 how interdisciplinarity may be useful to address the current gaps regarding the elements we describe about climate change, migration and health systems resilience.**

**We have also included a few sentences to describe the importance of distinguishing interdisciplinary from multidisciplinary.**


** **

- *As well as explaining more of the detail of Table 1 and Figure 1. (As an aside, in Table 1 I would propose there would be value in thinking about health system software as more than staff training, essentially; relationships among staff within the system and with the public also matter, for example).*

**A presentation and explanation have been provided in the article now.**

-  
*In terms of the earlier sections of the paper I was not sure why the first section is titled ‘climate migrants and health systems’, as the focus is on health challenges rather than health systems*. 

**The subtitle of this section has been changed to "climate migrants and health challenges".**

 

- * I also found that the logic and structure of the sections ‘climate change in the global health context’, and ‘health systems resilience in the climate change context’ made it difficult to follow the argument within them. In ‘climate change in the global health context’, this might be because the very tight referencing practice has overshadowed the argument.*

**We have reviewed the flow of this section, and the subtitle of this section has been changed to "climate migrants and health systems".**

 

- *In ‘health systems resilience in the climate change context’, the linkage between the different points presented is not very clear (i.e. the argument connecting them).*

**We have reviewed the flow of this section and changed its subtitle to emphasize the need to continue research on this concept, which is still a little too vague. The last section has been moved to become the first section on the need for interdisciplinarity.**

 

- *Some other minor points for review in p.3:* *What is an heuristic literature review?*

**A review of the non-systematic literature but which only includes useful articles on the subject and to develop our arguments. We made this clear in the correction.**

 

- *How are the 10 papers that were identified in the PubMed search used in the text, or is the point here that only 10 papers were identified?* 

**Yes, the point is that only 10 articles have been published, which shows how little is still written on the subject and the most relevant are cited in our article**

 

- *At the first mention, briefly clarify the significance of the Lancet Countdown for this paper.* 

**Thanks for this suggestion. We have clarified this significance in the text (p.7)**

 

- *One final comment: given that this is a very closely argued piece, there would be value in some really close copy editing – as, for example, missing words in sentences, long sentences, and sentences that are phrased quite clumsily, hinder understanding.*

**We had the latest version revised by a scientific editor, Donna Riley. However, for this re-submission, we have asked an additional native English speaker (Lara Schwarz) to review and edit the whole text.**
